# Population-Level Shifts in Cervical Cancer Stage Distribution in the United States Following Human Papillomavirus (HPV) Vaccine Implementation

**DOI:** 10.7759/cureus.118618

**Published:** 2026-10-26

**Authors:** Eva Koshy, Christina Son

**Affiliations:** 1 Radiation Oncology, University of Chicago, Chicago, USA

**Keywords:** cervical cancer, cervical cancer screening, health disparities, hpv vaccination, population-based study, seer database, stage migration

## Abstract

Objective: To assess whether human papillomavirus (HPV) vaccine introduction is associated with shifts in stage at cervical cancer diagnosis and to examine whether sociodemographic disparities and evolving imaging practices have influenced these observed trends.

Methods: We analyzed 103,831 cervical cancer cases diagnosed between January 2004 and December 2022 from the Surveillance, Epidemiology, and End Results (SEER) database. Stage at diagnosis was classified as non-distant or distant disease and compared across sociodemographic subgroups, including age group at diagnosis (<40 vs. ≥40 years), race/ethnicity, income, and urban-rural status. Temporal trends in distant-stage disease were evaluated using a linear probability model including year of diagnosis, age group, and an age group-by-year interaction term.

Results: Across the entire cohort (N = 103,831), 14,622 patients (14.1%) presented with distant-stage disease. Among patients <40 years, distant disease increased from 5.6% in 2004 to 9.2% in 2022, corresponding to an annual increase of 0.18 percentage points per year (95% CI 0.12-0.23, p < 0.001). Among patients ≥40 years, distant disease increased from 12.6% in 2004 to 20.4% in 2022, corresponding to an annual increase of 0.39 percentage points per year (95% CI 0.34-0.44, p < 0.001). The annual increase was 0.21 percentage points greater among patients ≥40 years (95% CI 0.14-0.29, interaction p < 0.001).

Conclusion: Over the past two decades, distant-stage cervical cancer increased in the United States, with a significantly steeper increase among women ≥40 years than among women <40 years. The comparatively smaller increase among younger women is consistent with a possible contribution of HPV vaccination at the population level; however, individual vaccination status was unavailable, and a causal relationship cannot be established. Persistent racial, socioeconomic, and geographic disparities in distant-stage presentation emphasize the ongoing need for equitable access to vaccination and screening.

## Introduction

Human papillomavirus (HPV) is the most common sexually transmitted infection worldwide and is the leading cause of cervical cancer [1-3]. The introduction of the HPV vaccine was a major advancement in public health, offering the potential to prevent the majority of HPV-related cancers [2,4-8]. Since its initial approval in 2006, the HPV vaccine has been widely integrated into immunization programs [3,5]. The rollout began with the quadrivalent and bivalent vaccines targeting HPV types 16 and 18, and later expanded to a vaccine that covers additional oncogenic types [4,9,10]. Today, in the United States, the Advisory Committee on Immunization Practices (ACIP) recommends routine vaccination for all adolescents at ages 11-12 [3,5].

Clinical trials and real-world studies have demonstrated the high efficacy of HPV vaccines, with 90-100% effectiveness in preventing persistent HPV 16/18 infection and high-grade cervical lesions among young women [2,9]. The nonavalent vaccine further provides over 95% efficacy against infection with additional high-risk HPV types [10]. Countries with high vaccination coverage, such as the United Kingdom and Australia, have reported up to 90% reductions in cervical cancer rates among vaccinated cohorts [11,12]. Long-term follow-up confirms that the vaccine's protection persists for at least 10-17 years post-vaccination, with no evidence of waning efficacy [13]. Importantly, population-wide vaccination also contributes to herd immunity, indirectly protecting individuals who are not vaccinated [2,11].

Despite proven efficacy, HPV vaccine uptake varies considerably across regions and populations [14,15]. In the United States, approximately 61% of adolescents aged 13-17 have completed the HPV vaccine series, but state-level coverage ranges from under 40% in Mississippi to over 84% in Rhode Island [14,15]. Urban areas and states in the Northeast tend to have higher coverage, while rural and Southern states have lower coverage [15,16]. Sociodemographic and geographic disparities are evident at the state, county, and ZIP code levels, with lower uptake in areas of higher poverty and among certain racial and ethnic groups [16-18].

While studies have shown that HPV vaccination reduces the overall incidence of cervical cancer, its effect on the stage at which cervical cancer is diagnosed is less well understood. Earlier-stage detection is associated with better outcomes, yet it remains unclear whether the introduction of the HPV vaccine has shifted the stage distribution, particularly among vaccinated age cohorts. Complicating this question is the phenomenon of stage migration, whereby advances in diagnostic imaging, such as the increased use of computed tomography (CT) and positron emission tomography (PET), enable more sensitive detection of metastatic disease. This can result in an apparent increase in distant-stage diagnoses over time, even in the absence of true changes in disease burden. Furthermore, it is unknown whether disparities in vaccine uptake across geographic regions or socioeconomic groups have affected the stage distribution of cervical cancer.

The HPV vaccine stands as a highly effective tool for cancer prevention, with robust evidence supporting its role in reducing HPV infections, precancerous lesions, and cervical cancer incidence [2,11,12]. However, disparities in vaccine uptake across geographic regions and socioeconomic groups remain a significant barrier to maximizing the public health benefits of HPV vaccination [15-18]. Addressing these disparities is essential for achieving the full potential of HPV vaccination and moving closer to the elimination of HPV-related cancers [16,19].

In this study, we assess whether HPV vaccine introduction is associated with shifts in stage at cervical cancer diagnosis, and examine the sociodemographic disparities and evolving imaging practices that may have influenced these observed trends.

## Materials and methods

Data source

The study was conducted at the University of Chicago, Chicago, Illinois, USA. Data were obtained from the Surveillance, Epidemiology, and End Results (SEER) database of the National Cancer Institute [20], which collects population-based information on cancer incidence, treatment, and survival across multiple U.S. regions. SEER represents approximately 45.9% of the U.S. population and includes data from 17 registries. The database provides detailed information on tumor site, histology, stage at diagnosis, demographic characteristics, and survival outcomes.

Study population

Patients were eligible for inclusion if they were aged 20 years or older and had a confirmed primary invasive carcinoma of the cervix uteri (International Classification of Diseases for Oncology, Third Edition (ICD-O-3) site code C53.0-C53.9) diagnosed between January 2004 and December 2022 [21]. The study period began in 2004 to provide a pre-vaccine baseline before HPV vaccine introduction in 2006 and to allow assessment of temporal trends as vaccine-eligible cohorts entered adulthood. The study period was also chosen to evaluate contemporary trends in stage distribution over nearly two decades. No additional exclusion criteria were applied. Stage classification was based on SEER Summary Stage to maintain consistency across the study period despite revisions in American Joint Committee on Cancer (AJCC) staging definitions that incorporated imaging findings beginning in 2018 [22].

Variables and covariates

The stage at diagnosis was dichotomized using SEER Summary Stage as non-distant or distant disease. Distant disease represented metastasis to remote organs, while cases not classified as distant were categorized as non-distant for the binary outcome used in the analyses.

Sociodemographic variables included age at diagnosis (<40 vs. ≥40 years), race (White, Black, Asian, American Indian, or Unknown individuals), ethnicity (Non-Hispanic vs. Hispanic), county-level median income (categorized as <$60,000, $60,000-$90,000, and >$90,000), and urban-rural residence classification (urban vs. rural). Urban-rural classification was derived from SEER county-level metropolitan status. Counties designated as metropolitan (≥1 million, 250,000-1 million, or <250,000 population) were classified as urban, whereas nonmetropolitan counties (adjacent and non-adjacent to metropolitan areas) were classified as rural. The <40 and ≥40-year age groups were selected to represent cohorts differing in potential HPV vaccine exposure. The quadrivalent HPV vaccine was introduced in 2006 and recommended for females aged nine to 26; patients diagnosed at age <40 years were increasingly more likely over the study period to have had the opportunity for vaccination during adolescence, whereas those ≥40 years largely reflect individuals who would have been ineligible for vaccination.

Statistical analysis

Trends in distant-stage disease were examined by year of diagnosis from 2004 to 2022. Descriptive statistics were generated to summarize patient and disease characteristics. Pearson chi-square tests were used for unadjusted comparisons of the proportion of patients presenting with distant-stage disease across categorical sociodemographic groups, including race, ethnicity, county income, and urban-rural residence. Temporal trends in distant-stage disease were evaluated using a linear probability model with distant versus non-distant-stage as the binary outcome. Year of diagnosis was modeled as a continuous variable, and the model included year of diagnosis, age group (<40 vs. ≥40 years), and an age group-by-year interaction term. The year coefficients estimated the annual absolute change in the proportion of distant-stage disease, expressed as percentage points per year. The interaction term was used to test whether temporal trends differed between age groups. Robust standard errors were used to calculate 95% confidence intervals and p-values. Subgroup analyses were conducted to evaluate trends across race, ethnicity, county income, and urban-rural classification.

Multivariable logistic regression was performed to identify predictors of distant-stage cervical cancer at diagnosis. The model included age at diagnosis (<40 vs. ≥40 years), race, ethnicity, year of diagnosis, county income category, and metropolitan residence as covariates, with reference categories defined a priori. Results are reported as odds ratios (OR) with 95% confidence intervals (CI). The statistical significance of individual regression coefficients was assessed using the Wald χ² test.

All statistical analyses were performed using IBM SPSS Statistics for Windows, Version 30 (Released 2024; IBM Corp., Armonk, New York, United States), and significance was defined as a two-sided p-value <0.05.

Ethics statement

This study used de-identified, publicly available data from the SEER database and was therefore exempt from Institutional Review Board review. No individually identifiable patient information was accessed, and written informed consent was not required.

## Results

A total of 103,831 patients with invasive cervical carcinoma diagnosed between January 2004 and December 2022 were identified in the SEER database. A total of 14,622 patients (14.1%) presented with distant-stage disease at diagnosis (Table 1).

**Table 1:** Patient characteristics

Variable	Category	<40 years n (%)	≥40 years n (%)	Total N
Race	White	23,305 (79.0%)	54,617 (73.5%)	77,922
	Black	3,554 (12.1%)	11,718 (15.8%)	15,272
	Asian	1,796 (6.1%)	6,687 (9.0%)	8,483
	American Indian	301 (1.0%)	541 (0.7%)	842
	Unknown	533 (1.8%)	779 (1.0%)	1,312
Ethnicity	Non-Hispanic	19,954 (67.7%)	55,577 (74.8%)	75,531
	Hispanic	9,535 (32.3%)	18,765 (25.2%)	28,300
Income	< $60,000	4,750 (16.1%)	12,460 (16.8%)	17,210
	$60,000–$90,000	18,889 (64.1%)	46,048 (61.9%)	64,937
	> $90,000	5,846 (19.8%)	15,821 (21.3%)	21,667
	Unknown	4 (0.0%)	13 (0.0%)	17
Urbanicity	Urban	26,271 (89.1%)	65,766 (88.5%)	92,037
	Rural	3,158 (10.7%)	8,508 (11.4%)	11,666
	Unknown	60 (0.2%)	68 (0.1%)	128
Stage	Non-distant	27,417 (93.0%)	61,792 (83.1%)	89,209
	Distant	2,072 (7.0%)	12,550 (16.9%)	14,622
Year of Diagnosis	2004–2013	15,209 (51.6%)	38,304 (51.5%)	53,513
	2014–2022	14,280 (48.4%)	36,038 (48.5%)	50,318
Total Cohort	All patients	29,489 (100%)	74,342 (100%)	103,831

Racial and sociodemographic disparities were observed. Black women had a higher proportion of distant-stage disease than White women (16.9% vs. 13.8%, p < 0.001) and Asian women (16.9% vs. 13.0%, p < 0.001). American Indian women had a distant-stage rate of 14.3%. By ethnicity, non-Hispanic patients had higher rates of distant disease than Hispanic patients (15.1% vs. 12.6%). Socioeconomic gradients were evident: patients in lower-income counties (<$60,000) were more likely to present with distant-stage disease than those in higher-income counties (>$90,000; 14.9% vs. 13.7%, p = 0.018). Patients residing in rural regions likewise had higher distant-stage rates than those in urban metropolitan areas (15.8% vs. 14.0%, p < 0.001).

The proportion of patients presenting with distant-stage disease increased over the study period, while the proportion with non-distant disease correspondingly decreased (Figure 1).

**Figure 1:**
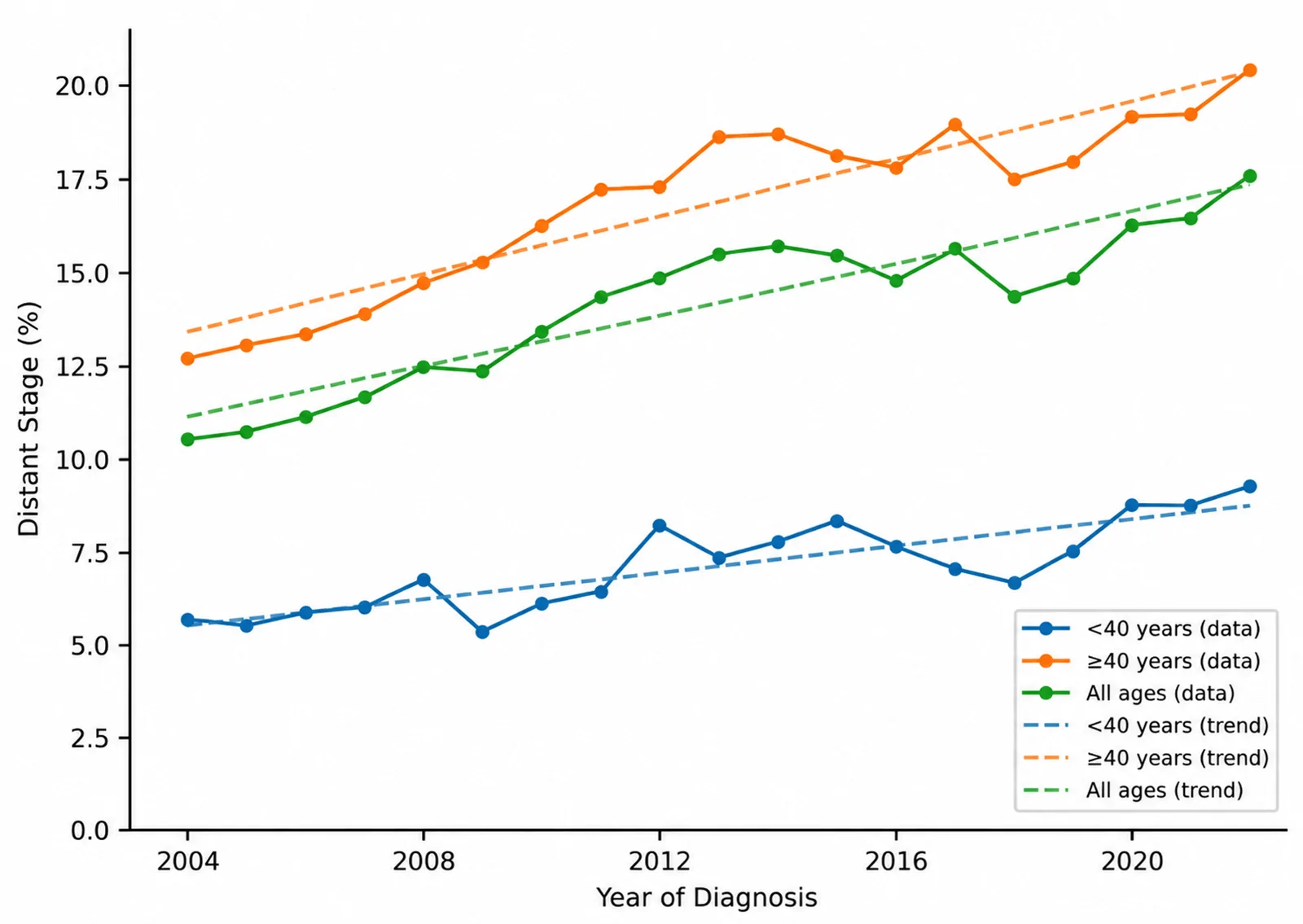
Distant disease in cervical cancer by age group (2004–2022)

When stratified by age at diagnosis, the proportion of distant disease increased from 5.6% in 2004 to 9.2% in 2022 among patients aged <40 years. In the temporal trend model, this corresponded to an annual increase of 0.18 percentage points per year (95% CI 0.12-0.23, p < 0.001). Among patients aged ≥40 years, distant disease increased from 12.6% in 2004 to 20.4% in 2022, corresponding to an annual increase of 0.39 percentage points per year (95% CI 0.34-0.44, p < 0.001). The annual increase was 0.21 percentage points greater among patients aged ≥40 years (95% CI 0.14-0.29, interaction p < 0.001), demonstrating a significantly steeper temporal trend in the older age group (Figure 1).

Age-stratified subgroup analyses were also performed by race and county income (Figure 2) and by ethnicity and urban-rural residence (Figure 3).

**Figure 2:**
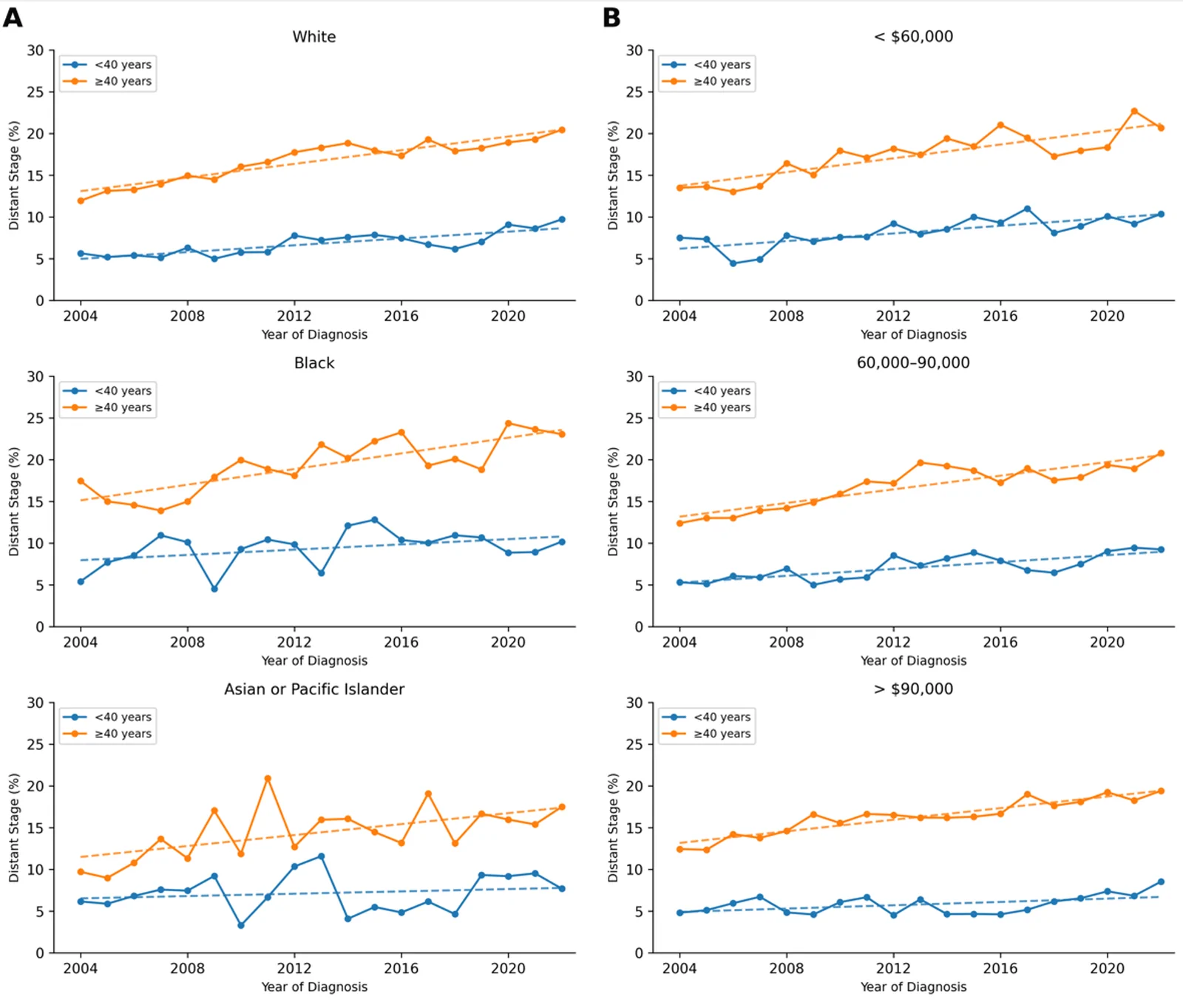
Age-stratified trends in distant-stage cervical cancer A: race; B: county income

**Figure 3:**
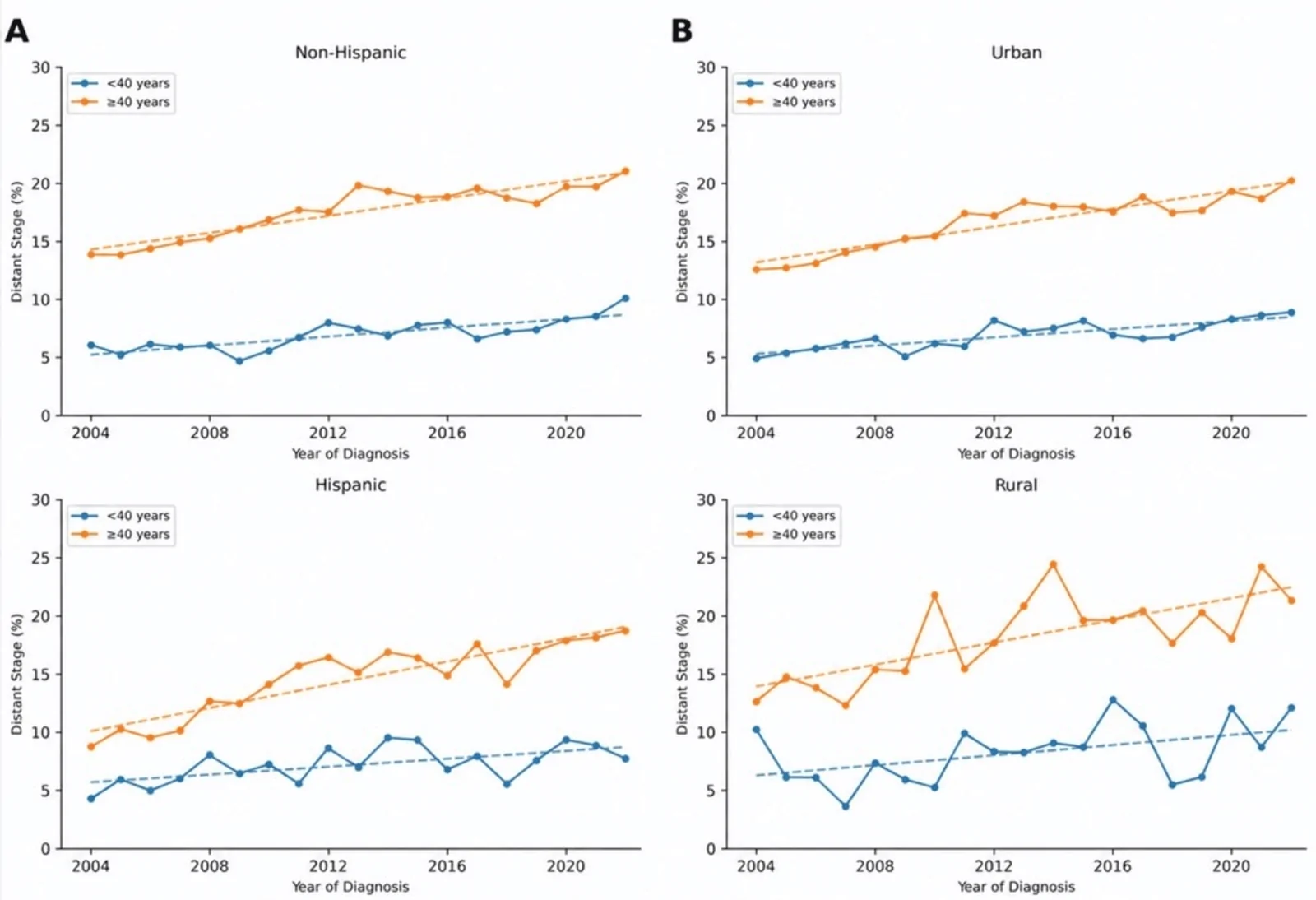
Distant disease in cervical cancer by ethnicity and urban-rural residence (2004–2022) A: ethnicity; B: urban-rural residence

Overall, rates of distant-stage cervical cancer remained lower among patients <40 years across racial, ethnic, socioeconomic, and geographic subgroups.

In contrast, patients ≥40 years demonstrated a consistent increase in distant-stage presentation across all examined subgroups, with similar temporal trends. These subgroup analyses demonstrate that the temporal increase in distant-stage cervical cancer is predominantly age-dependent and consistent across population subgroups.

In multivariable logistic regression, age remained the strongest predictor of distant-stage cervical cancer at diagnosis (Table 2).

**Table 2:** Multivariable logistic regression for odds of distant-stage cervical cancer at diagnosis Adjusted odds ratios were estimated using multivariable binary logistic regression. All variables shown were mutually adjusted. Statistical significance of individual regression coefficients was assessed using the Wald χ² test. OR: odds ratio; CI: confidence interval

Characteristics	Adjusted OR	95% CI	Wald χ²	p-value
Race				
White	Reference	—	—	—
Black	1.17	1.11–1.23	38.37	<0.001
Asian	0.84	0.78–0.90	25.81	<0.001
American Indian	1.03	0.84–1.27	0.09	0.767
Unknown	0.29	0.22–0.38	80.18	<0.001
Ethnicity				
Non-Hispanic	Reference	—	—	—
Hispanic	0.86	0.82–0.90	44.85	<0.001
Age group				
<40 years	Reference	—	—	—
≥40 years	2.65	2.52–2.78	1533.73	<0.001
Year of diagnosis				
2004–2013	Reference	—	—	—
2014–2022	1.31	1.26–1.36	219.46	<0.001
County median income				
<$60,000	Reference	—	—	—
$60,000–$90,000	0.98	0.93–1.04	0.44	0.507
>$90,000	0.93	0.88–1.00	4.1	0.043
Unknown	0.43	0.05–3.54	0.62	0.432
Urbanicity				
Urban	Reference	—	—	—
Rural	1.06	1.00–1.13	3.63	0.057
Unknown	1.06	0.59–1.91	0.04	0.850

Women aged ≥40 years had significantly higher odds of presenting with distant disease compared with women younger than 40 years (adjusted OR = 2.65, 95% CI 2.52-2.78), independent of race, ethnicity, year of diagnosis, income category, and metropolitan residence. Racial and ethnic disparities persisted after adjustment, and higher income remained associated with lower odds of distant-stage disease.

## Discussion

Summary of main results

In this population-based analysis of over 103,000 women with invasive cervical cancer diagnosed between 2004 and 2022, we observed an increasing proportion of distant-stage disease over time. The increase was significantly steeper among women aged ≥40 years than among women aged <40 years. In multivariable logistic regression, age ≥40 years was the strongest independent predictor of distant-stage disease at diagnosis (adjusted OR = 2.65, 95% CI 2.52-2.78). Persistent racial, socioeconomic, and geographic disparities in stage at diagnosis were also observed, with Black women, patients in lower-income counties, and rural patients more likely to present with distant-stage disease.

Results in the context of published literature

Prior studies have demonstrated substantial population-level reductions in HPV infection, precancerous lesions, and cervical cancer incidence following HPV vaccine implementation. Long-term follow-up from the PATRICIA trial and Nordic cohort studies confirmed durable protection against high-risk HPV types and precancerous lesions [9,13]. Furthermore, real-world data from highly vaccinated countries such as the United Kingdom and Australia demonstrated up to 90% reductions in cervical cancer and cervical intraepithelial neoplasia grade 3 incidence among vaccinated cohorts [11,12]. In our study, the comparatively smaller increase in distant-stage presentation among younger women is consistent with a possible contribution of HPV vaccination at the population level. However, because individual vaccination status was unavailable in SEER and age at diagnosis served only as a proxy for potential vaccine exposure, these findings cannot establish a direct effect of vaccination on stage at diagnosis.

Several mechanisms could potentially contribute to the observed age-related differences, including HPV vaccination. Vaccination prevents infection with high-risk HPV types 16 and 18, among others [23]. A changing relative distribution of HPV-associated and HPV-independent cervical cancers may also contribute, as HPV-independent tumors appear to have more aggressive clinical behavior [24]. However, SEER does not include tumor-level HPV status, so this could not be evaluated directly. Evidence that specific non-vaccine HPV types have a greater tendency for distant metastasis remains limited. In addition, vaccinated cohorts may differ in their participation in routine screening. Ssentongo et al. found that individuals who received the HPV vaccine were significantly more likely to undergo cervical cancer screening than unvaccinated women, with the effect strongest among those who received all three doses [25]. Herd immunity effects at the population level may also reduce transmission of high-risk HPV types [11]. These factors could potentially contribute to the observed differences between age groups, but the ecological design of this study does not permit determination of the independent contribution of vaccination. The increasing use of PET/CT and other advanced imaging over the study period likely contributed to stage migration and may explain a portion of the observed increase in distant-stage disease.

The age-related divergence observed in this study parallels national incidence and mortality trends. Shahmoradi et al. reported that declines in cervical cancer incidence between 2001 and 2019 occurred among women younger than 40 years, while older women experienced stable or increasing rates [6]. Similarly, Dorali et al. found reductions in cervical cancer mortality among women younger than 25 years during the vaccine era [7]. Together with modeling studies suggesting that vaccination and screening can accelerate the timeline toward elimination [19,26], our findings demonstrate population-level temporal patterns occurring during the HPV vaccine era but do not establish vaccination as the cause of the observed differences in stage at diagnosis.

Despite overall gains, our study highlights persistent inequities in stage at diagnosis. Black women and those residing in lower-income or rural areas were more likely to present with distant-stage disease, patterns consistent with previous reports of disparities in both HPV vaccine uptake and screening access [8,16-18]. Spencer et al. and Niccolai et al. demonstrated that racial and socioeconomic factors remain strong predictors of vaccination initiation [16,18], while Alarid-Escudero et al. showed that state-level disparities in vaccine coverage and screening intensity could delay elimination timelines by decades [8].

Strengths and limitations

The primary strength of this study is its use of a large, population-based dataset of over 103,000 patients with a nearly two-decade follow-up period, providing substantial statistical power to detect temporal trends in distant-stage disease. The use of a consistent non-distant versus distant outcome allowed uniform analysis across the study period despite changes in AJCC staging definitions. The inclusion of multiple sociodemographic subgroups allowed for a thorough characterization of disparities. Age stratification at the 40-year threshold provided a clinically meaningful proxy for potential vaccine exposure.

This study has several limitations. The SEER database lacks individual-level data on HPV vaccination history or cervical cancer screening, precluding direct assessment of vaccination status as a predictor. Age at diagnosis therefore serves only as a proxy for potential vaccine exposure, and the observed population-level associations cannot establish a causal effect of vaccination on stage at diagnosis. There is also no data available on the use of advanced imaging, including CT or PET, which may have contributed to the observed stage migration through improved detection of metastatic disease. Additionally, we cannot account for partial vaccination or catch-up immunization, nor for changes in screening guidelines over the study period that may also have influenced stage at diagnosis.

Implications for practice and future research

These findings demonstrate different temporal patterns in distant-stage cervical cancer across age groups with differing opportunities for HPV vaccine exposure. The comparatively smaller increase among younger women is consistent with a possible contribution of vaccination at the population level, but individual vaccination status was unavailable, and the present analysis cannot establish that vaccination attenuated the increase in distant-stage disease. Broader adoption of PET/CT imaging and improved diagnostic staging may also have contributed to the observed temporal patterns. Addressing racial, socioeconomic, and geographic disparities through targeted outreach, community-based vaccination programs, and equitable screening infrastructure remains essential to reducing the cervical cancer burden [8,16-18]. Future research incorporating individual-level vaccination, screening, and imaging data will be necessary to more directly evaluate the factors contributing to temporal changes in distant-stage presentation.

## Conclusions

Distant-stage cervical cancer increased in the United States between 2004 and 2022, with a significantly steeper temporal increase among women aged ≥40 years compared with women aged <40 years. Age ≥40 years was the strongest independent predictor of distant-stage disease at diagnosis. These population-level findings demonstrate an age-related divergence in temporal trends that is consistent with a possible contribution of HPV vaccination, but a direct effect cannot be established because individual vaccination status was not available in the database. Racial, socioeconomic, and geographic disparities in stage at presentation persisted throughout the study period.
